# Role of High-Resolution Ultrasonography With Colour and Duplex Doppler in the Evaluation of Acute Scrotal Diseases

**DOI:** 10.7759/cureus.49231

**Published:** 2023-11-22

**Authors:** Satyanarayana Kummari, Saraswata Das, Suvanya Mahajan

**Affiliations:** 1 Radiology, Great Eastern Medical School & Hospital, Srikakulam, IND; 2 Department of Radiodiagnosis, College of Medicine and JNM Hospital, Kalyani, IND; 3 Department of Radiology, Government Medical College, Jammu, Jammu, IND

**Keywords:** high-resolution ultrasonography, colour and duplex doppler ultrasonography, perineal abscess, fournier's gangrene, varicocele, testicular torsion, orchitis, epididymitis, epididymo-orchitis, acute scrotum

## Abstract

Introduction

The term 'acute scrotum' denotes the sudden initiation of pain in the scrotal region. Acute scrotum is a frequent medical condition in children and adults. Ultrasound facilitates precise diagnosis and differentiation of numerous causes of acute scrotum. The objective of our research was to assess the utility of ultrasonography in the identification and prevalence determination of causes of acute scrotum.

Materials and methods

Patients with acute scrotal pain referred to the Department of Radiodiagnosis at Great Eastern Medical School & Hospital (GEMS), Srikakulam, India, were added in the research. This is a prospective observational study. High-frequency linear transducer (4-15 MHz) was used for imaging. Grey scale, colour and duplex Doppler were performed routinely. The ultimate diagnosis was established by considering the clinical results, follow-up observations, intraoperative observations and, when accessible, histopathological analysis. For the statistical analysis, IBM SPSS Statistics for Windows, version 22 (released 2013; IBM Corp., Armonk, New York, United States). was used. Descriptive analysis was conducted. The Kendall rank correlation coefficient was used to evaluate the non-parametric association between side and torsion.

Results

A total of 120 patients were included for statistical analysis. Inflammatory pathology was the most common cause of acute scrotum, followed by testicular torsion and torsion of testicular appendage. Testicular torsion and side of pain did not show a significant association.

Conclusion

High-resolution ultrasonography with colour and duplex Doppler sonography is an excellent imaging modality exhibiting exceptional sensitivity and specificity for the accurate diagnosis of acute scrotal diseases. Inflammatory pathology was the most common cause of acute scrotum, followed by testicular torsion and torsion of the testicular appendage.

## Introduction

Acute scrotum refers to an abrupt onset of pain in the scrotum, which may or may not be accompanied by redness, tenderness and enlargement of the scrotum. Both paediatric and adult patients frequently present with acute scrotal pain. Acute scrotum can arise from a multitude of causes, spanning from benign and self-limiting conditions to critical medical and surgical emergencies [1-4]. Acute scrotal pain can be attributed to various aetiologies, including infection, inflammatory conditions, such as acute epididymitis, acute epididymo-orchitis and acute orchitis; additional conditions, including testicular abscess, scrotal wall cellulitis, funiculitis, Fournier's gangrene and perineal abscess; vascular conditions, including torsion of testis or testicular appendage; varicocele; testicular trauma; hernia; ureteric calculus; and, infrequently, testicular neoplasms [1-3]. Early differentiation between the surgical and nonsurgical causes of acute scrotal pain is of utmost significance, as prompt surgical interventions in cases of spermatic cord torsion can effectively preserve the testicular function [5].

Distinguishing between several clinical scenarios that may manifest as acute scrotal pain poses a considerable diagnostic challenge in the field of clinical medicine. Furthermore, it is worth noting that the laboratory and physical examination results in these settings can sometimes exhibit similarities. This, together with the possibility of patient guarding and lack of cooperation, might lead to a restricted and non-specific physical examination. Establishing a definitive diagnosis can often be challenging when relying just on clinical history and physical examinations, thus necessitating the use of imaging techniques for this reason.

Ultrasound imaging facilitates precise discrimination among several aetiologies contributing to acute scrotal pain. Ultrasound is quick, inexpensive, easily accessible and easily reproducible, allowing for immediate assessment of scrotal emergencies. Computed tomography (CT) scans subject the testicles to radiation. Conversely, magnetic resonance imaging (MRI) is not readily accessible, limiting its availability for diagnostic purposes. Recent developments in high-resolution grey-scale imaging techniques have led to significant advancements in the field. These include the introduction of tissue harmonic imaging. The application of tissue harmonic imaging, colour and duplex Doppler ultrasound has significantly broadened the utilization of ultrasound technology in the evaluation of causes of acute scrotum. High-resolution ultrasound continues to be the preferred imaging modality due to its exceptional sensitivity and specificity in the diagnosis of acute scrotal diseases, including acute epididymitis or testicular torsion [6,7]. The objective of our research was to assess the utility of ultrasonography in the identification and prevalence determination of the underlying causes in patients with acute scrotum who sought ultrasound examination at the radiology department.

## Materials and methods

All the individuals who presented to the Department of Radiodiagnosis at Great Eastern Medical School and Hospital (GEMS), Srikakulam, India, for an ultrasound and colour Doppler examination of the scrotum with acute onset pain, including or excluding swelling, redness, and tenderness of the scrotum, were added in the research. Before the examination, the patients gave their informed and signed assent. The research was granted approval by the Institutional Human Ethics Committee (IHEC) of GEMS (approval no. IHEC/GEMS/2021/45). This study was conducted between July 2021 and December 2022 at GEMS. The research conducted was a prospective observational study that took place in a tertiary hospital setting. Ultrasound examination was performed using a Mindray DC-80 X-Insight ultrasound machine (Mindray, Shenzhen, China) using a 4-15 MHz frequency linear probe. Grey-scale real-time imaging, as well as colour and duplex Doppler, were performed as a part of the standard procedure. The ultrasound procedure was conducted by a radiologist possessing six years of professional expertise. The ultimate diagnosis was established by considering the clinical results, follow-up observations, intraoperative discoveries and, when accessible, histopathological analysis. The research excluded patients with a documented history of scrotal injuries, who did not provide informed consent, and those who were lost to follow-up.

Imaging technique

Ultrasound of the scrotum was conducted with the patient lying in a supine posture, wherein the scrotum is adequately supported either by a folded towel positioned between the thighs or a rolled towel placed over the thighs. The penis was repositioned in an upward direction and thereafter concealed using a cloth. A high-frequency, linear transducer with a range of 4-15 MHz was used to capture the images. Typically, scanning was performed using a high-frequency transducer. However, in cases involving abrupt enlargement of the scrotum, edema of the scrotum or large hydrocele, a lower-frequency transducer may be required to ensure a sufficient imaging depth. A comparison was made between the testes and epididymides in terms of their size and echogenicity. The assessment also included an examination of the anatomical structures located in the spermatic cord and the scrotal skin thickness. The optimization of colour Doppler parameters was undertaken to enhance the detection capability of low flow velocities. Spectral Doppler was employed to detect intratesticular arterial flows, while power Doppler was utilized to identify slow flows. For unilateral symptoms, grey-scale and Doppler parameters were optimised on the normal side before comparing. Supplementary techniques, such as assuming a standing position and performing the Valsalva manoeuvre, were employed in the assessment of a varicocele.

Statistical analysis

The collected data were imported into a Microsoft Excel 2010 spreadsheet (Microsoft, USA), and statistical analysis was conducted with the help of IBM SPSS Statistics for Windows, version 22 (released 2013; IBM Corp., Armonk, New York, United States).

## Results

**Figure 1:**
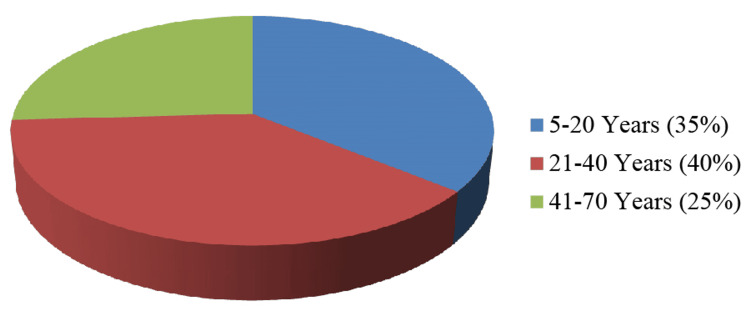
Age distribution

**Figure 2:**
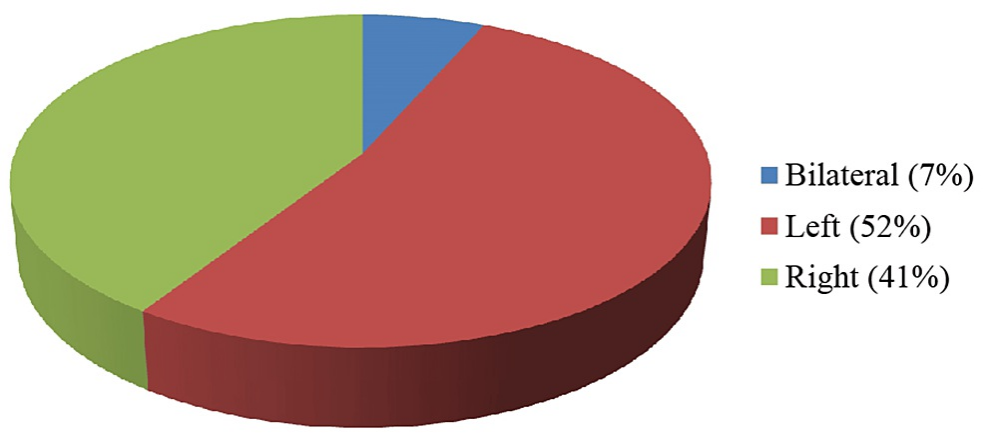
Side of pain

A total of 134 patients who had abrupt onset of pain in the scrotum were sent to the Department of Radiodiagnosis for scrotal sonography. Eight patients with a documented history of trauma and six patients got away while being followed up on. Therefore, they were eliminated from the analysis. The rest of the 120 patients were analyzed statistically. The age of the patients in our research varied between five and 70 years, with a mean age of 26.5±15.5 years. Most of the patients fell between the age range of 21-40 years (Figure 1). The occurrence of pain on the left side of the scrotum was observed in 52% of the patients (Figure 2).

Inflammatory pathology was the most common cause of acute scrotal pain (40%), followed by torsion of the testis (18.3%) and torsion of the testicular appendage (10%) (Table 1). The side of the testicle and the occurrence of testicular torsion did not show a significant association (R = 0.1; P = 0.3). The study findings revealed a higher incidence of inflammatory pathology among those aged 31 to 50 years, while occurrences of torsion of testis and its appendage were predominantly reported in individuals below the age of 20. The prevalence of varicocele was highest among those aged 31 to 40 years (Table 2).

**Table 1:** Causes of acute scrotal pain

Diagnosis	Frequency	Percentage
Ureteric calculus	6	5.00
Fournier’s gangrene	4	3.33
Funiculitis	12	10.00
Hernia	3	2.50
Inflammatory pathology	48	40.00
Normal	6	5.00
Perineal abscess	4	3.33
Torsion of testis	22	18.33
Torsion of testicular appendage	12	10.00
Testicular tumour	1	0.83
Varicocele	2	1.66
Total	120	100

**Table 2:** Etiology of acute scrotum with age group

Age group	05-20 years	21-30 years	31-40 years	41-50 years	51-60 years	61-70 years
Ureteric calculus	0	1	4	1	0	0
Fournier’s gangrene	0	0	0	1	3	0
Funiculitis	1	2	4	3	2	0
Hernia	0	0	0	1	2	0
Inflammatory pathology	15	7	14	7	4	1
Normal	1	2	1	1	1	0
Perineal abscess	0	0	1	2	1	0
Torsion of tes​tis	14	4	3	1	0	0
Torsion of testicular appendage	12	0	0	0	0	0
Testicular tumour	0	0	1	0	0	0
Varicocele	0	0	2	0	0	0
Total	43	16	30	17	13	1

## Discussion

The occurrence of acute scrotal pain is a frequently encountered issue in children and adults. Acute scrotum is characterised by an abrupt onset of pain in the scrotum, which may or may not be accompanied by the enlargement, redness and tenderness of the scrotum. Although bilateral occurrences are occasionally documented, unilateral cases predominate [1-4]. Frequent aetiologies of acute scrotal pain encompass infections; inflammatory conditions, such as acute epididymitis, acute epididymo-orchitis and acute orchitis; additional conditions, including testicular abscess, scrotal wall cellulitis, funiculitis, Fournier's gangrene and perineal abscess; vascular conditions, including torsion of testis or testicular appendage; varicocele; testicular trauma; hernia; ureteric calculus; and, infrequently, testicular neoplasms [1-3]. Distinguishing between surgical and nonsurgical aetiologies of acute scrotal pain is of utmost importance, as early interventions for spermatic cord torsion can save the testes [5]. The utilization of ultrasound imaging facilitates precise identification and discrimination of various aetiologies contributing to acute scrotal pain. Ultrasound is rapid, affordable, easily accessible and easily reproducible, allowing for the immediate assessment of scrotal emergencies. High-resolution ultrasonography using colour and duplex Doppler imaging techniques continues to be the recommended imaging modality because of its excellent sensitivity and specificity for the diagnosis of acute scrotal diseases. The current research found that the age at which the highest number of acute scrotum cases occurs is typically between 21 and 40 years (Figure 1). Approximately 52% of patients with acute scrotum were observed on the left side (Figure 2).

**Figure 3:**
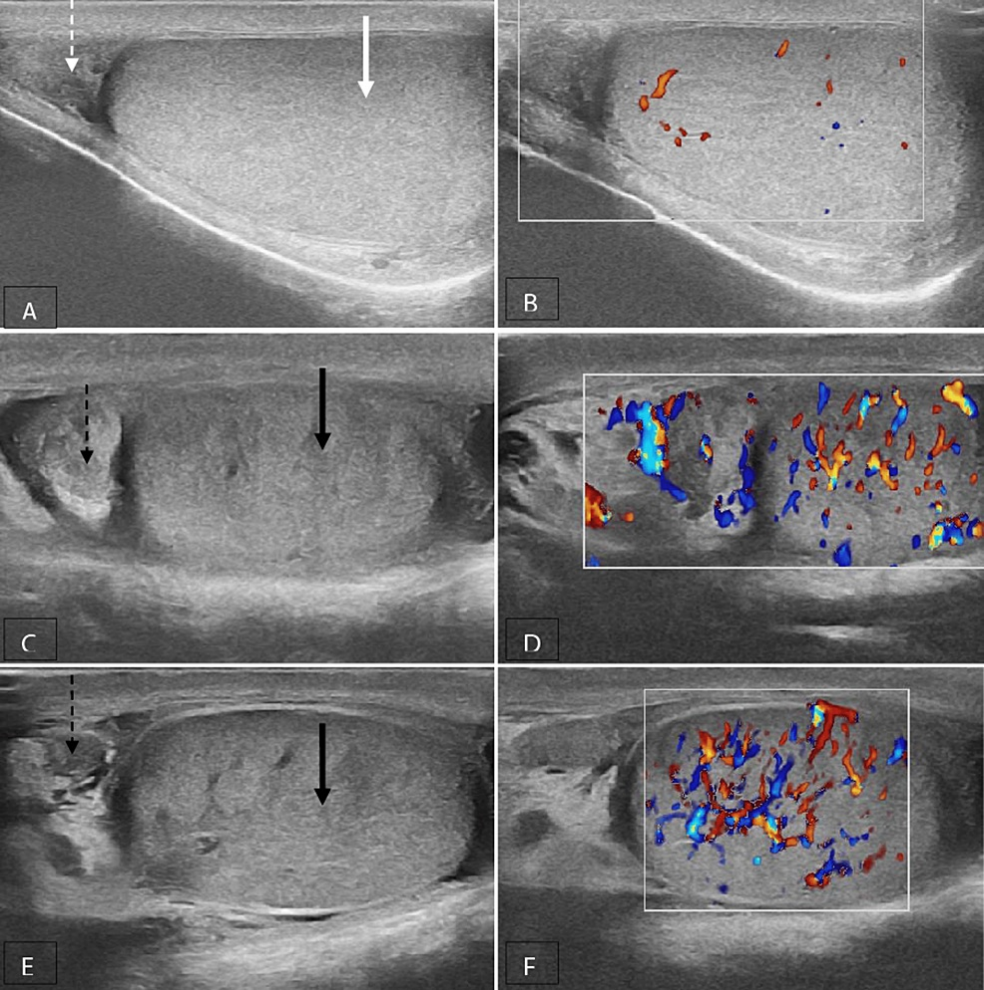
Acute epididymo-orchitis A, B Grey-scale and colour Doppler images of a normal right testis (solid white arrow) and epididymis (dotted white arrow); C, D, E, F Grey-scale and color Doppler images of the left testis (solid black arrow) and epididymis (dotted black arrow), which are enlarged in size and show heterogeneous echotexture with increased vascularity.

**Figure 4:**
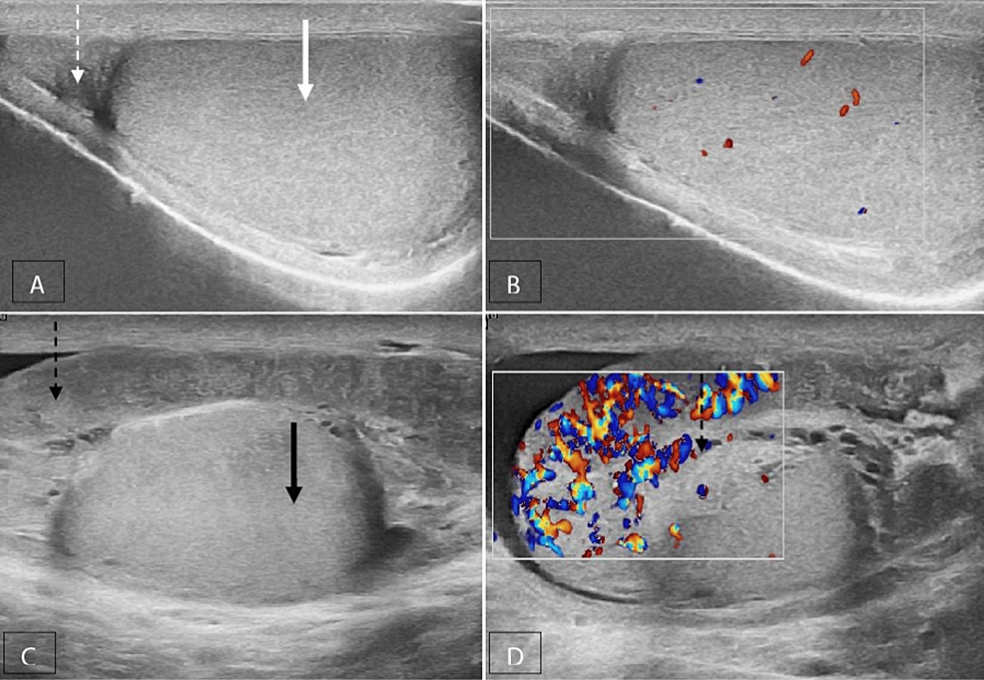
Acute epididymitis A, B Grey-scale and colour Doppler images of a normal right testis (solid white arrow) and epididymis (dotted white arrow); C, D grey-scale and colour Doppler images of the left testis (solid black arrow) and epididymis (dotted black arrow). The left testis is normal. The left epididymis is enlarged in size and shows heterogeneous echotexture with increased vascularity.

**Figure 5:**
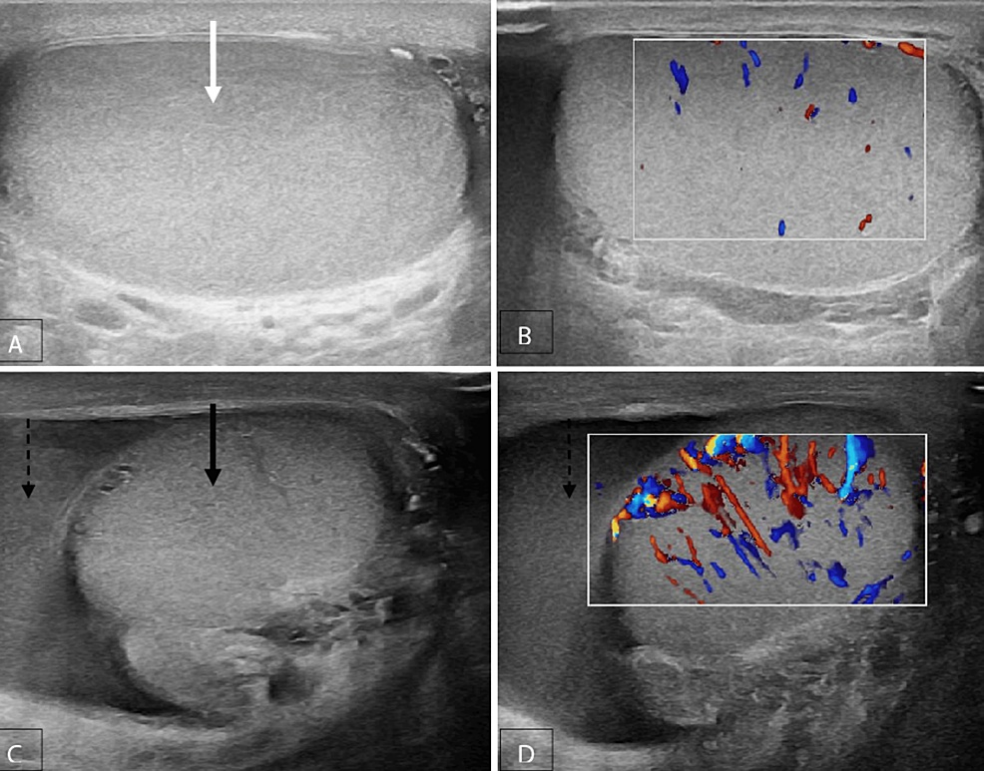
Acute orchitis with a reactive hydrocele A, B Grey-scale and colour Doppler images of a normal left testis (solid white arrow); C, D grey-scale and colour Doppler images of the right testis (solid black arrow) that is enlarged in size and shows a heterogeneous echotexture with increased vascularity and reactive hydrocele (dotted black arrow).

**Figure 6:**
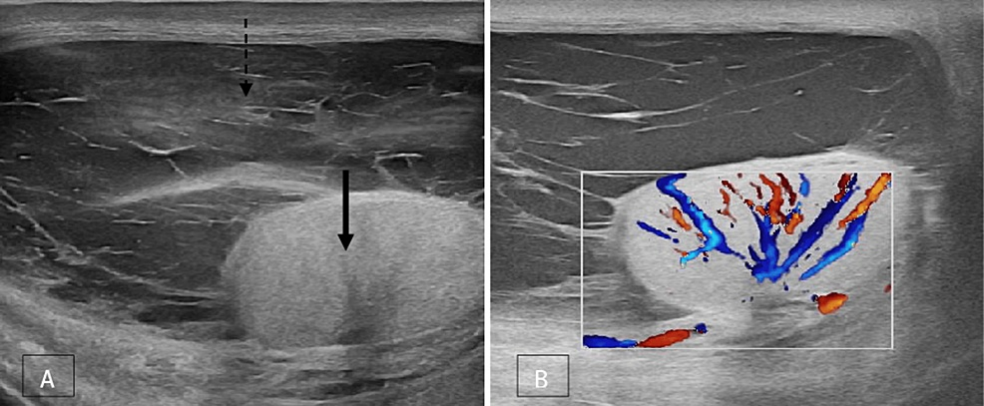
Acute orchitis with pyocele A, B Grey-scale and colour Doppler images of the right testis (solid black arrow) shows a heterogeneous echotexture with increased vascularity and pyocele (dotted black arrow).

Inflammatory diseases, such as acute epididymo-orchitis (Figure 3), acute epididymitis (Figure 4) and acute orchitis (Figures 5, 6), are the most frequent aetiologies of acute scrotum in both children and adults. The ultrasound characteristics observed in cases with acute epididymo-orchitis encompass decreased echogenicity of the testis and epididymis, enlarged size of the testis and epididymis, presence of secondary hydrocele and scrotal wall thickening. The colour Doppler examination revealed the presence of increased vascularity, high flow and low resistance pattern. The most common cause of orchitis is the contiguous spread from the epididymis. Primary orchitis is a relatively uncommon condition that is predominantly attributed to the mumps virus, with bilateral involvements observed in 14-35% of cases [6]. The age at which the highest number of cases occurs is typically between 40 and 50 years [8].

**Figure 7:**
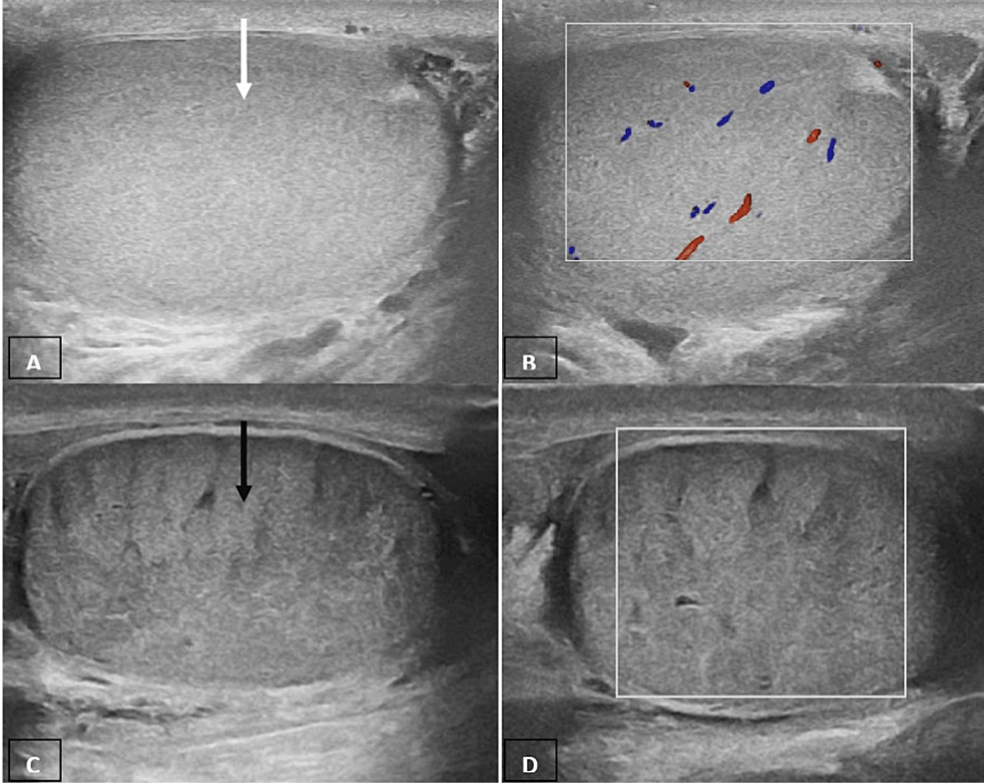
Acute torsion A, B Grey-scale and colour Doppler images of a normal right testis (solid white arrow); C, D grey-scale and colour Doppler images of the left testis (solid black arrow) that shows a heterogeneous echotexture and no vascularity.

We found that inflammatory pathology was most common in those aged 31 to 50 years (Table 2). The current research found that inflammatory pathology was responsible for 40% of all cases of acute scrotum and was the most common cause of acute scrotal pain (Table 1). It was also the most common cause of acute scrotal pain in children (Table 2). Other authors' research also showed that acute epididymo-orchitis was the most frequent aetiology of acute scrotum [9,10]. Although there are numerous potential aetiologies for acute scrotum, testicular torsion (Figure 7) is among the most crucial.

The testes have limited tolerance to ischemia, making them unable to endure prolonged periods without enough blood supply. Failure to promptly diagnose testicular torsion can lead to irreversible testicular damage and subsequent loss of testicular function. Testicular torsion, also known as the twisting of the spermatic cord, involves the initial obstruction of venous flow, followed by arterial flow. The severity of testicular ischemia is contingent upon the magnitude of torsion ranging from 180° to 720° and the duration of the twisting. In the initial stages of torsion (one to three hours), ultrasound examination reveals normal testicular echogenicity. Enlargement of the afflicted testis and decreased or heterogeneous echogenicity are common observations with progressions. It is possible for the orientation of the testis, epididymis and cord to be inverted. A conclusive determination of a total testicular torsion is established when the presence of blood flow is observed on the unaffected side, while its absence is observed on the affected side. The term 'incomplete torsion' is used to describe a condition where the twisting of the spermatic cord is less than 360°, resulting in some arterial blood flows remaining in the affected testis. In these instances, it is imperative to conduct a thorough examination of the two testes through the utilization of transverse views in order to make a meticulous comparison. The presence of normal echogenicity accompanied by a mild testicular enlargement is indicative of a favourable prognosis, suggesting survivability. Conversely, the presence of considerable enlargement, heterogeneous echotexture and scrotal wall hypervascularity are indicative of testicular infarction. Testicular torsion is predominantly observed in paediatric and adolescent populations, but it has the potential to manifest across all age groups [8,11].

In the current research, the highest incidence of testicular torsion (Figure 7) was reported among those below the age of 20 years (Table 2). Approximately 52% of the patients with acute scrotum were observed on the left side. Khaleghnejad-Tabari et al. have also reported a similar observation [12]. The recorded prevalence of testicular torsion among adolescent males is estimated to be approximately one case per 4000 individuals on an annual basis, constituting 25% of acute scrotum cases [2]. Cavusoglu et al. and Mushtaq et al. have reported a prevalence of testicular torsion at rates of 29% and 20% of acute scrotum cases, respectively [9,13]. Similar results to those reported by Mushtaq et al. were also seen in our study. The current research found that testicular torsion was responsible for 18.3% of all cases of acute scrotum, and it was the second most common cause for acute scrotum (Table 1). The side of the testicle and the occurrence of testicular torsion did not show a significant association.

The most frequent aetiology of acute scrotum in children is the torsion of the testicular appendage [14-16]. The ultrasound characteristics of torsion of the testicular appendage include the presence of a lesion with reduced echogenicity with the central hypoechoic area adjacent to the epididymis. The occurrence of torsion of the testicular appendages constitutes a significant proportion, ranging from 24% to 46% of acute scrotum cases. The clinical presentation closely resembles that of testicular torsion. The purpose of ultrasound in this disease is to rule out testicular torsion and acute epididymo-orchitis [17]. The condition is frequently seen in children among the ages of seven and 13 years [1].

**Figure 8:**
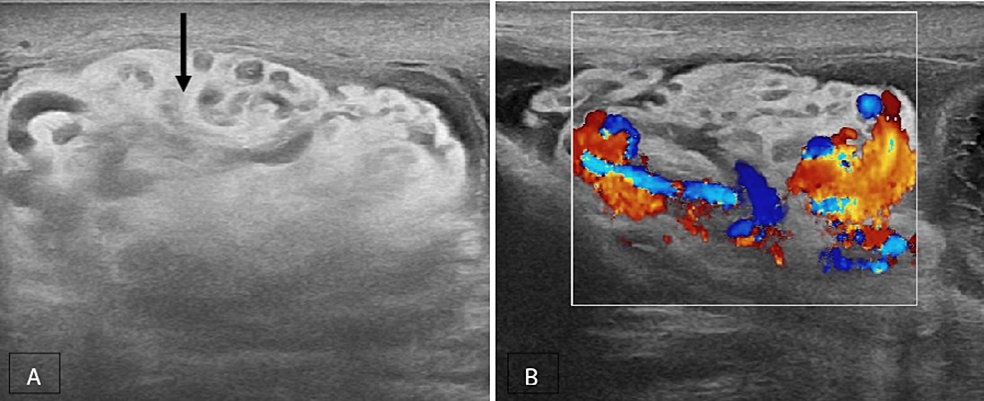
Funiculitis A, B Grey-scale and colour Doppler images of the right spermatic cord (solid black arrow) show enlarged, edematous, echogenic and heterogeneous spermatic cord with increased vascularity.

In the current research, torsion of the testicular appendages is responsible for 10% of all cases of acute scrotum, and it was the third most frequent aetiology for acute scrotum (Table 1). Occasionally, the torsion of the testicular appendage may not be visualized on ultrasound imaging. Funiculitis (Figure 8) is characterized by inflammation of the spermatic cord. It typically presents as a painful lump in the inguinal region with pain that may radiate to the scrotum. Ultrasound shows an enlarged, edematous and heterogenous spermatic cord (Figure 8).

In addition, there is evidence of soft-tissue stranding and a mass-like appearance of the echogenic fat within a distended inguinal canal. Furthermore, hyperemia is present and does not show any change upon application of the Valsalva manoeuvre [18,19]. Moreover, concurrent occurrences of epididymitis or epididymo-orchitis, pyocele or scrotal abscess are possible [19]. In the current research, funiculitis is responsible for 10% of all cases of acute scrotum, and it was the third most frequent aetiology for acute scrotum (Table 1). The prevalence was higher among individuals aged 31 to 50 years (Table 2).

The presence of pain in the testis or scrotum may also be attributed to the referral of pain from visceral or somatic structures. Referred pain through the genitofemoral nerve may cause the pain to radiate to the testes. The presence of calculus or other pathological conditions in the upper ureter might result in referred pain to the testis, while distension in the lower ureter can lead to scrotal pain on the same side [20]. In the current research, calculus was responsible for 5% of all cases of acute scrotum (Table 1). Specifically, these cases were attributed to ureteric and vesicoureteric junction calculus.

**Figure 9:**
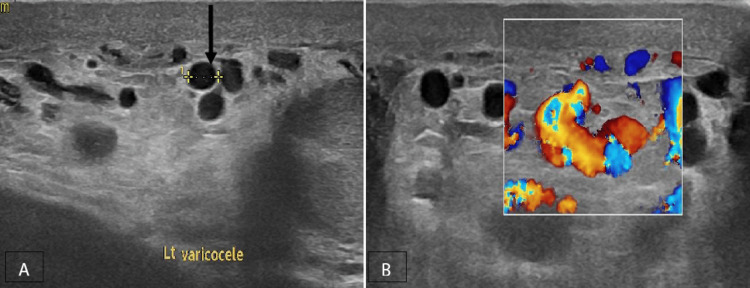
Varicocele A, B Grey-scale and colour Doppler images of the left side shows multiple dilated pampiniform plexus of veins with reflux (solid black arrow).

In our research, it was shown that 5% of the patients of acute scrotum did not exhibit any sonographic abnormalities in the scrotal structures. A scrotal ultrasonography examination that just focuses on the scrotum has the potential to overlook life-threatening infections or perineal abscess. The imaging features of a perineal abscess consist of a clearly delineated collection characterised by varying internal echogenicities resulting from its contents, accompanied by fluctuating peripheral blood flow and adjacent hyperemia. The presence of a more widespread infection raises concern for Fournier's gangrene, a urological emergency characterised by polymicrobial necrotizing fasciitis, which has been associated with fatality rates nearing 75% [21]. The diagnostic features of Fournier's gangrene include the presence of intrascrotal gas, which is characterised by several echogenic foci exhibiting a ring-down or reverberation artefact. It is imperative to distinguish this condition from the presence of air in a bowel containing inguinal hernia, which can be accomplished by demonstrating the gas peristalsing in the bowel lumen. In the present study, the occurrence of perineal abscess and Fournier's gangrene constituted 3.3% each of the total cases with acute scrotum. They were more common among the 41-60-year age group. In our analysis, we observed that strangulated hernia, varicocele (Figure 9) and testicular tumour were less frequently identified as causes of acute scrotum, accounting for 2.5%, 1.6% and 0.83% of cases, respectively.

There are some limitations inherent in our research. A limited sample size of patients was studied. It was a short-duration study. The patients excluded from our study were those with a documented history of trauma and those who were lost to follow-up. However, the implementation of more comprehensive research endeavours with a larger cohort would yield a more precise depiction of the matter under investigation.

## Conclusions

Ultrasound facilitates precise diagnosis and differentiation of numerous causes of acute scrotal pain. Ultrasound is quick, cost effective, portable and easily repeatable and allows for a rapid evaluation of scrotal emergencies. We conclude that high-resolution ultrasonography with colour and duplex Doppler sonography is a highly effective imaging modality exhibiting exceptional sensitivity and specificity for the accurate diagnosis of acute scrotal diseases. Inflammatory pathology was the most common cause of acute scrotal pain, followed by torsion of the testis and torsion of the testicular appendage. The most common age group for acute epididymo-orchitis was 31-50 years, that of testicular torsion was five to 20 years and that of the torsion of the testicular appendage was five to 20 years.
